# Sexual behaviors and access to HIV services during the COVID-19 pandemic among cisgender men who have sex with men in Lima, Peru

**DOI:** 10.1186/s12889-025-23886-8

**Published:** 2025-08-22

**Authors:** Kai Vu, Hugo Sánchez, Robinson Cabello, José Hidalgo, Sayan Dasgupta, Ann Duerr, Alexander Lankowski

**Affiliations:** 1 Vaccine & Infectious Disease Division, Fred Hutchinson Cancer Center, Seattle, WA USA; 2 School of Public Health, University of Washington, Seattle, WA USA; 3 Epicentro, Lima, Peru; 4 Vía Libre, Lima, Peru; 5 Department of Global Health, University of Washington, Seattle, WA USA

**Keywords:** MSM, COVID-19, Sexual behaviors, Peru, HIV prevention, Sex-on-premises venue

## Abstract

**Background:**

The COVID-19 pandemic significantly impacted sexual behaviors, access to health services, and other factors related to HIV vulnerability among sexual and gender minority populations globally. This study investigates such changes among men who have sex with men (MSM) in Lima, Peru.

**Methods:**

We analyzed data from a cross-sectional survey that was conducted initially in 2018–2019 (‘pre-pandemic’ period, *n* = 382) and then repeated in 2020–2021 (‘mid-pandemic’ period, *n* = 387). The survey asked about participants’ sexual behaviors in the previous three months, including attendance of sex-on-premises venues (SOPVs) and the use of online platforms to meet partners, as well as their knowledge and behaviors related to HIV testing and prevention. We assessed for differences in sexual behaviors and HIV testing/prevention knowledge between the mid-pandemic period and the pre-pandemic period using robust Poisson regression, including in multivariable models adjusting for age and educational attainment. The mid-pandemic survey included additional questions asking about access to HIV services and changes in perceived health status during the pandemic, which we analyzed descriptively.

**Results:**

Participants in the mid-pandemic period were significantly less likely to report they had attended an SOPV, met a sex partner online, engaged in group sex, or had three or more partners in the past three months. However, the prevalence of other HIV-related sexual risk behaviors, including condomless anal sex and substance use in a sexual context, was unchanged compared to pre-pandemic. Among mid-pandemic survey participants, SOPV attendance and meeting a partner online were both associated with a range of sexual risk behaviors, similar to the relationship observed between these behaviors during the pre-pandemic period.

**Conclusions:**

We observed relatively modest differences in the prevalence of sexual risk behaviors during, versus prior to, the COVID-19 pandemic. These findings underscore the importance of minimizing disruptions to HIV prevention and sexual health services for vulnerable populations such as MSM in Peru.

**Supplementary Information:**

The online version contains supplementary material available at 10.1186/s12889-025-23886-8.

## Background

The COVID-19 pandemic had a profound impact on many aspects of daily life for sexual minority populations such as men who have sex with men (MSM), including sexual behaviors [1–6]. Pandemic-related disruptions in access to HIV prevention and care services, as well as changes in sexual behaviors associated with increased HIV vulnerability, have been described in a variety of settings [1, 7–11], threatening to further exacerbate long-standing health inequities experienced by these groups. Changes in sexual behaviors documented in previous studies have ranged from increased sex abstinence to greater reliance on the use of online dating apps to meet sex partners, owing to physical distancing measures and lockdowns [7, 12]. As a result of economic challenges related to the COVID-19 pandemic [9, 13, 14], MSM may increasingly turn to sex work or other forms of transactional sex [9, 15]. Studies also observed an increase in psychological distress and depressive symptoms among MSM, which may be linked to a greater likelihood of engaging in HIV-associated risk behaviors, including transactional sex [8].

In Peru, HIV disproportionately affects MSM, with an estimated prevalence of over 10%, compared to an overall population prevalence of less than 1% [16]. Heteronormative culture, homophobia, and HIV-associated stigma are cited as some of the largest barriers to accessing HIV and related sexual health services [17–19]. Peru was also particularly impacted by the COVID-19 pandemic, with among the highest per capita mortality rates in the world despite its strict initial lockdown and prolonged period of highly restrictive social distancing measures lasting well into the third year of the pandemic [20, 21]. A recent study suggests that lockdown restrictions resulted in decreased past-year and quarterly HIV testing in Peru [22]. In addition to disrupting existing HIV testing and treatment services, the COVID-19 pandemic may have played an important role in delaying the rollout of HIV pre-exposure prophylaxis (PrEP) in Peru, which became available via the public healthcare system in mid-2023 [23].

Studies conducted with MSM in Lima prior to the COVID-19 pandemic found that attendance of sex-on-premises venues (SOPVs, e.g., saunas, hourly hotels, bars/discos with dark rooms) was common, and associated with sexual behaviors such as group sex, transactional sex, and sex under the influence of alcohol [24]. The use of online platforms to meet sex partners was also shown to be extremely common and associated with higher rates of engagement in sexual risk behaviors in this setting [25]. The implementation of strict social distancing measures, lockdowns, and other public health interventions to prevent COVID-19 transmission resulted in the closure of many venues used for meeting and having sex with partners, including SOPVs. This likely raised the need for alternate means of meeting partners, such as through online platforms like geosocial networking (GSN) apps, and may have resulted in other behavioral changes [26, 27]. However, the extent to which the COVID-19 pandemic altered patterns of SOPV attendance and online partner seeking among MSM in Peru – and how the pandemic may have influenced HIV risk and prevention behaviors – is not well documented.

In this study, we assessed the potential impact of the COVID-19 pandemic on sexual behaviors, as well as access to HIV services, among MSM in Peru by analyzing data from a repeated cross-sectional survey. Additionally, we aimed to describe how the pandemic impacted self-perceived mental, physical, and sexual health in this population.

## Methods

### Study design and participants

We conducted a repeated, cross-sectional, internet-based survey among MSM in Lima, Peru. Our primary analytic objective was to compare the prevalence of HIV-related risk and prevention behaviors – including SOPV attendance and the use of online platforms to meet sex partners – among MSM in Lima during and prior to the COVID-19 pandemic. As a secondary objective, we evaluated the interrelationships between SOPV attendance, online partner seeking, and sexual behaviors during the COVID-19 pandemic, and how this may have changed compared to pre-pandemic. Additionally, we aimed to describe the impact of the COVID-19 pandemic on access to HIV services and self-perceived mental, physical, and sexual health.

The original survey was carried out from November 2018 to May 2019 (‘pre-pandemic’ survey). The primary analysis of the pre-pandemic survey – which evaluated the relationship between SOPV attendance, the use of online platforms to meet sex partners, and HIV risk and prevention behaviors – has previously been published [24]. The survey was then repeated from November 2020 to September 2021 (‘mid-pandemic’ survey), a period corresponding to the third major wave of COVID-19 cases in Peru [28]. In both instances, participants were recruited by disseminating the online survey link via social media platforms affiliated with Epicentro, a community-based organization in Lima dedicated to LGBTQ+ health promotion. Participation was anonymous and no incentive was provided. For the mid-pandemic survey, we replicated the same online outreach strategy using the same dissemination platforms as in the initial survey, with the goal of obtaining a second independent sample from the same target population and sampling frame. Eligible participants included in this analysis were adults aged 18 years or older who identified as cisgender MSM. The survey was designed for use on both desktop and mobile devices. An initial landing page displayed the consent form, instructions, and eligibility criteria. Participants had to provide a digital attestation of their informed consent and conformity with the eligibility criteria prior to entering the survey. The study received ethical approval from the Vía Libre Comité Institucional de Bioética (Lima, Perú) and the University of Washington Institutional Review Board (Seattle, USA).

### Survey instrument

The survey was administered in Spanish and implemented using REDCap [29]. The core set of 19 questions that appeared on both the pre-pandemic and mid-pandemic survey has previously been described in detail in the context of the primary analysis of the pre-pandemic survey [24]. Briefly, this included questions on socio-demographics, sexual partners and behaviors in the last three months (e.g., attendance of SOPVs and use of online platforms), HIV testing history (including self-reported HIV status and current use of antiretroviral therapy [ART], if living with HIV), and knowledge of HIV prevention methods such as pre-exposure prophylaxis (PrEP) and “undetectable equals untransmissible” (U = U). The mid-pandemic survey included an additional 12 to 18 questions (depending on one’s responses) on how the COVID-19 pandemic had affected participants’ lives, including finances, living situation, self-perceived health, sexual behaviors and relationships, access to HIV treatment and prevention services, concerns about HIV acquisition (if HIV-negative) or management (if HIV-positive), as well as history of sexually transmitted infection (STI) in the last three months. To maintain a similar survey length across settings, we omitted several sub-questions on the mid-pandemic survey that had appeared on the original pre-pandemic survey but were deemed less relevant in the context of the COVID-19 pandemic (e.g., related details of the specific SOPVs participants reported attending).

### Measures

All predictor and outcome variables included in regression analyses were operationalized as binary variables, except for age, which was included as a continuous variable in our multivariable models. We defined SOPV attendance as reporting that one had either met a sex partner, or had sex at, any of the following types of venues in the previous three months: sauna, hotel, sex club, pornographic movie theater, or bar/disco. Online platform use was defined as reporting that one had met at least one sex partner via an online platform in the previous three months. Condomless anal sex (CAS) was based on whether participants reported engaging in CAS with either their last or penultimate partner in the previous three months. Sexualized substance use – defined as use before or during a sexual encounter in the previous three months – was assessed for the following: alcohol, marijuana, poppers, cocaine, ecstasy, amphetamines, heroin, ketamine, and LSD. In regression analyses, this was operationalized as four separate binary variables corresponding to ‘any substance’ and for each of the three most prevalent specific substances reported (alcohol, poppers, and marijuana).

### Statistical analysis

Data analysis and visualization was performed in Stata version 18.0 [30] and RStudio version 2023.12.1.402 [31]. We used descriptive statistics to summarize socio-demographic characteristics, self-reported HIV status, and challenges reported by participants in accessing HIV services. Differences in socio-demographic factors by survey period (pre-pandemic vs. mid-pandemic) were assessed using the two-sample t-test for continuous variables and the chi-squared test for categorical variables. For the mid-pandemic survey, five-point Likert scale items – which asked about the impact of the COVID-19 pandemic on self-perceived health status, HIV risk and prevention behaviors, and level of concern about HIV acquisition/management – were summarized as frequencies and proportions.

For our primary analytic objective, we used Poisson regression with robust standard errors to estimate crude and multivariable prevalence ratios (PR) and 95% confidence intervals (CI) for the association between survey period (mid-pandemic vs. pre-pandemic) and reported sexual behaviors as well as variables related to HIV testing/prevention history. All multivariable models included continuous age and dichotomous educational attainment (+/- university education) as covariables. PR estimates represent the prevalence of a given factor of interest in the mid-pandemic survey relative to the prevalence in the pre-pandemic survey.

For our secondary objective, among mid-pandemic survey participants only, we fit separate sets of robust Poisson regression models estimating the association between each of our two explanatory variables of interest (SOPV attendance and online platform use) and dependent variables of interest (sexual behaviors, HIV testing/prevention history). Crude and multivariable PR and 95% CI represent the prevalence of a given factor of interest among SOPV attendees relative to non-attendees, or among online platform users relative to non-users. To assess for differences in the effect size of these relationships between the pre-pandemic and mid-pandemic survey periods, we added an interaction term by survey period (mid-pandemic vs. pre-pandemic) to these same models and then repeated the regression analyses, this time including the combined study population inclusive of both the pre-pandemic and mid-pandemic samples. Due to the large number of estimates evaluated for each of our primary and secondary analytic objectives, we used the Benjamini-Hochberg procedure [32] with a false discovery rate (FDR) of 0.05 to adjust for multiple comparisons.

Finally, we performed several sensitivity analyses to test the robustness of our findings across strata and to assess for potential confounding or effect modification based on two key variables (sexual orientation and self-reported HIV status) for which the observed prevalence was notably different between the mid-pandemic and pre-pandemic survey samples. We generated separate stratified models, by HIV status (positive vs. not positive) or by sexual orientation (bisexual vs. not bisexual), to estimate the association between survey period and factors of interest in each stratum. To assess the robustness of our findings to differences in the date of one’s survey participation during the COVID-19 pandemic (given that mid-pandemic participants were recruited over the course of 10+ months), we performed an additional sensitivity analysis in which mid-pandemic participants were binned into three discrete subgroups according to their survey response date (period 1: November 17, 2020 to December 31, 2020; period 2: January 1, 2021 to July 31, 2021; period 3: August 1, 2021 to October 1, 2021) and then performed the regression analyses separately for each subgroup. Date ranges for the three groups were determined based on temporal trends in COVID-19 incidence in Peru, with a notably higher incidence observed during period 2 [28]. Based on the significant differences we observed in the proportion of individuals living with HIV and identifying as bisexual in the mid-pandemic versus pre-pandemic sample (Table 1), a post-hoc sensitivity analysis was conducted, adjusting for these variables as well as for U = U knowledge given its potential as a proxy for health literacy.

## Results

Overall, 769 cisgender MSM completed either the pre-pandemic or mid-pandemic survey and were eligible for inclusion in this analysis. Of these, 382 participated in the initial pre-pandemic survey between November 26, 2018, and May 16, 2019 [24], and 387 participated in the mid-pandemic iteration of the survey from November 17, 2020, through September 30, 2021 (Supplementary Fig. 1, Additional File 1).

### Participant characteristics

Of the 387 MSM in the mid-pandemic survey, 87% identified as homosexual, 12% as bisexual, and 1% as heterosexual. The median age was 32 (interquartile range [IQR] 28–40), and approximately half had attained a university degree or higher. Overall, 94% of participants reported they had ever been tested for HIV and 39% self-identified as HIV-positive, all but one of whom indicated they were currently on ART. Compared to the pre-pandemic survey, participants in the mid-pandemic survey were, on average, slightly older, less likely to identify as bisexual, and more likely to be living with HIV (Table 1, Panel A).

**Table 1:** Demographic characteristics of the analysis population and barriers to HIV services during the COVID-19 pandemic (*N* = 769)

	Pre-pandemic survey participants, *N* = 382		Mid-pandemic survey participants, *N* = 387	*p*-value^a^
**Panel A. Participant characteristics**				
Age (years), median (IQR)	30 (25–37)		32 (28–40)	< 0.01
Monthly income (Soles), median (IQR)	1500 (700–3000)		1200 (60–3000)	0.44
Highest Education, n (% of N)				0.07
Partial secondary school (or less)	5 (1%)		3 (< 1%)	
Completed secondary school	144 (38%)		115 (30%)	
Completed technical/vocational school	55 (14%)		67 (17%)	
Completed university	131 (34%)		133 (34%)	
Obtained post-graduate degree	47 (12%)		68 (18%)	
Sexual Orientation, n (% of N)				0.01
Homosexual	297 (78%)		335 (87%)	
Bisexual	70 (18%)		46 (12%)	
Heterosexual	13 (3%)		5 (1%)	
Other	2 (< 1%)^b^		1 (< 1%)^c^	
Living Situation, n (% of N)				< 0.01
Lives alone	61 (16%)		82 (21%)	
Lives with friends/roommates	26 (7%)		37 (10%)	
Lives with family AND partner	0 (0%)		12 (3%)	
Lives with family only (not partner)	242 (63%)		212 (55%)	
Lives with partner only (not family)	53 (14%)		44 (11%)	
HIV-positive	102 (27%)		150 (39%)	< 0.01
Currently taking ART (among those HIV-positive)	96/102 (94%)		149/150 (99%)	
**Panel B. Barriers to HIV testing and treatment access related to the COVID-19 pandemic among mid-pandemic survey participants**				
		*HIV-positive and on ART, N = 149 *		
Reported ANY pandemic-related barrier to accessing HIV treatment, n (% of N)		49 (33%)		
Difficulty accessing ART refills		7 (5%)		
Difficulty with transport to clinic		12 (8%)		
Difficulty accessing ancillary services		38 (26%)		
Difficulty scheduling an appointment		3 (2%)		
Other difficulty accessing HIV care		7 (5%)^d^		
		*HIV-negative, N = 237*		
Reported ANY pandemic-related barrier to accessing HIV testing, n (% of N)		111 (47%)		
Limited availability of HIV testing services		64 (27%)		
Difficulty with transport to HIV testing site		57 (24%)		
Other difficulty accessing HIV testing		3 (1%)^e^		

*IQR* interquartile range, *ART* antiretroviral therapy.

a. Calculated using chi-squared test (continuous) and two-sample-t-test (categorical)

b. *n* = 2 “Other” includes “Pansexual” (2)

c. *n* = 1 “Other” includes “Demisexual” (1)

d. *n* = 7 “Other” difficulties reported: insurance (1), unemployment (1), long appointment times (1), tested positive for COVID-19 (2)

e. *n* = 3 “Other” difficulties reported: did not want to test (1), traveling (1), did not have money (1)

Mid-pandemic survey participants reported a median of 2 (IQR 1–5) sexual partners in the last three months, with 65% reporting they used an online platform to meet a partner and 55% reporting they attended a SOPV to meet a partner or have sex in the last three months. 44% reported engaging in CAS, 38% reported having a non-HIV STI diagnosis, and 43% reported any sexualized substance use in the last three months. Although 14% reported being paid for sex at least once in the last three months, only 2% identified as a sex worker. Relative to the three-month period immediately prior to the COVID-19 pandemic, 33% of participants reported a decrease in monthly income, 27% reported an increase, and the remaining 40% reported no change in income.

### Barriers to accessing HIV services during the COVID-19 pandemic

Among participants in the mid-pandemic survey who identified as HIV positive, 33% reported encountering any type of challenge accessing HIV care related to the pandemic, while 47% of HIV negative participants reported any type of challenge accessing HIV testing (Table 1, Panel B). Of the 237 mid-pandemic participants who were not living with HIV, 33 (14%) reported they had ever used PrEP, 21 (9%) reported they were currently on PrEP, and 14 (6%) reported they had ever received HIV post-exposure prophylaxis (PEP). Notably, close to half of ever PrEP users (42%, 14/33) reported either a temporary or ongoing interruption in their PrEP use due to COVID-19 pandemic-related barriers.

### Change in self-perceived health status, sexual behaviors, and HIV-related concerns among mid-pandemic participants

**Fig. 1:**
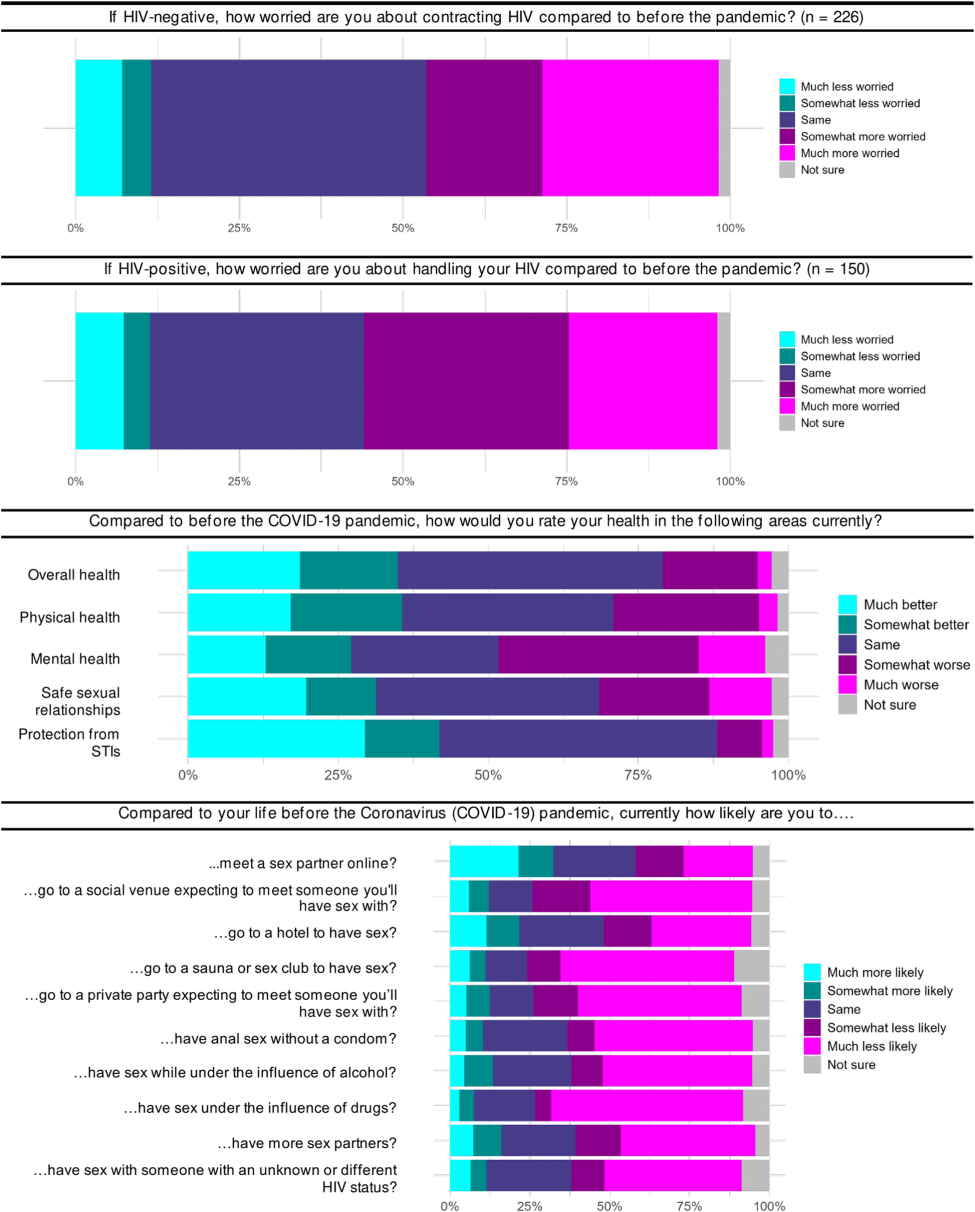
Change in self-perceived health status, sexual behaviors, and concerns about HIV acquisition or management compared to before the pandemic (N=387)

Over half (54%) of those identifying as HIV-positive reported they were more worried about managing their HIV compared to before the COVID-19 pandemic, and nearly half (45%) of HIV-negative participants reported they were more worried about acquiring HIV compared to pre-pandemic (Fig. 1).

The proportion who rated their self-perceived health as worse than before the pandemic varied depending on the domain being assessed: 18% of participants reported their overall health was worse, 27% for physical health, and 44% for mental health. In contrast, 35%, 19%, and 17% reported their overall, physical, and mental health, respectively, as better than before the pandemic. About one-quarter (28%) of participants reported they felt less able to have safe and healthy sexual relationships and 9% felt less able to protect oneself from STIs compared to before the pandemic. Half or more of participants reported they were less likely, compared to before the pandemic, to have sex with someone of an unknown or different HIV status, meet a sex partner at a social venue, engage in CAS, have sex under the influence of alcohol or drugs, or have more sex partners.

### Sexual behaviors, HIV testing history, and HIV prevention knowledge reported by mid-pandemic versus pre-pandemic survey participants

**Fig. 2:**
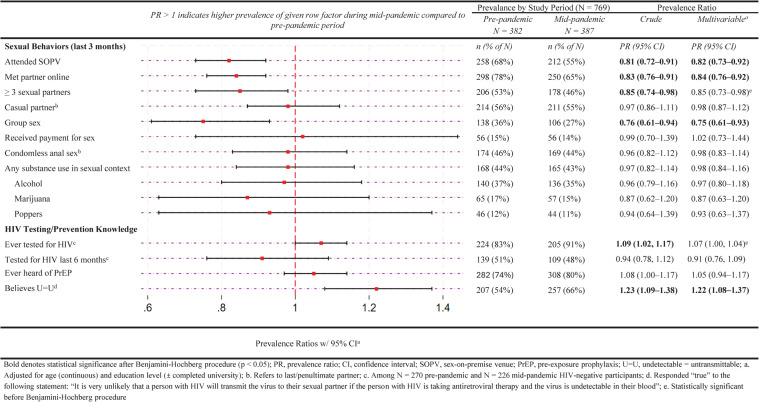
Prevalence of sexual behaviors and HIV testing/prevention knowledge comparing the mid-pandemic versus pre-pandemic survey period (*N* = 769)

Compared to the pre-pandemic survey, the observed prevalence among mid-pandemic survey participants was numerically lower for nearly all of the HIV-related sexual risk behaviors we assessed (Fig. 2).

Mid-pandemic participants were significantly less likely to report they had attended an SOPV (55% vs. 68%), met a sex partner online (65% vs. 78%), or engaged in group sex (27% vs. 36%) in the previous three months, but they were more likely to know about the concept of U = U (66% vs. 54%). Among those without an existing HIV diagnosis, there was no significant difference in HIV testing (ever or in the last 6 months) based on multivariable analysis. Stratifying by HIV status (Supplementary Table 1, Additional File 1) or by sexual orientation (Supplementary Table 2, Additional File 1) did not reveal substantial differences between stratum-specific estimates and the original unstratified estimates. No appreciable change in magnitude or statistical significance of our estimates was observed when adjusting for HIV status and sexual orientation in our multivariable models, nor when we additionally adjusted for U = U knowledge in these models (Supplementary Table 8, Additional File 1). Additionally, we did not observe a marked difference in our estimates across the three subgroups of mid-pandemic survey participants based on survey response date (Supplementary Table 7, Additional File 1).

### Factors associated with SOPV attendance among mid-pandemic survey participants

**Fig. 3:**
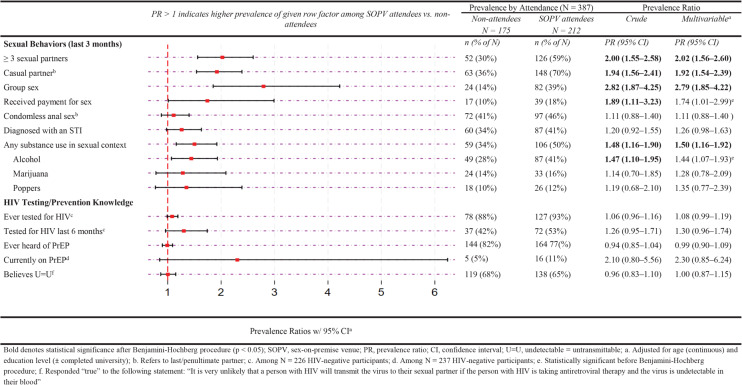
Factors associated with SOPV attendance among MSM in Lima during the COVID-19 pandemic (*N* = 387)

Among mid-pandemic survey participants, 55% (212/387) reported they had attended an SOPV in the last three months. Compared to non-attendees, those who attended an SOPV were significantly more likely to report they had three or more sexual partners, a recent casual partner, group sex, or any sexualized substance use in the previous three months (Fig. 3).

When stratified by HIV status (Supplementary Table 3, Additional File 1) or by sexual orientation (Supplementary Table 4, Additional File 1), stratum-specific estimates were not substantially different from the overall combined estimates.

### Factors associated with the use of online platforms to meet a sex partner among mid-pandemic survey participants

**Fig. 4:**
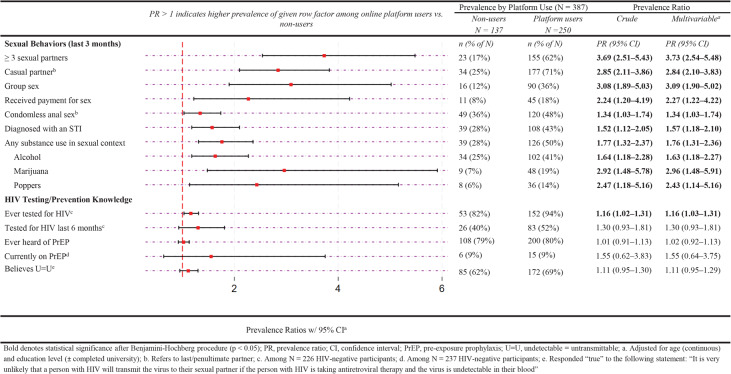
Factors associated with online platform use to meet a sex partner among MSM in Lima during the COVID-19 pandemic (*N* = 387)

Among mid-pandemic survey participants, 65% (250/387) reported meeting a sex partner online in the last three months. Those who met a partner online were significantly more likely to report they engaged in all of the sexual behaviors we evaluated in this analysis, as well as having ever tested for HIV (Fig. 4).

When stratified by HIV status (Supplementary Table 5, Additional File 1) or by sexual orientation (Supplementary Table 6, Additional File 1), the direction and magnitude of estimates were generally similar across strata.

### Interaction by survey period with respect to the association of SOPV attendance or online platform use with sexual behaviors

When we assessed for differences between the mid-pandemic and pre-pandemic survey in the relationship between SOPV attendance and sexual behaviors, alcohol use in a sexual context was the only factor for which we identified significant interaction by survey period, with a weaker association during the mid-pandemic period compared to the pre-pandemic period (PR_interaction_ 0.57, p_interaction_ = 0.03). For all other factors, there was no significant difference (p_interaction_ >0.05) by survey period in the effect size for SOPV attendance. For the relationship between online platform use and sexual behaviors, we identified only two factors for which there was significant interaction by survey period, both indicating a stronger association during the mid-pandemic period relative to pre-pandemic: reporting three or more partners (PR_interaction_ 2.34, p_interaction_ < 0.01) or reporting a casual sex partner (PR_interaction_ 1.77, p_interaction_ < 0.01) in the last three months.

## Discussion

Among MSM in Lima, Peru, we observed a significantly lower prevalence of certain sexual behaviors (SOPV attendance, online platform use, number of sex partners, and group sex) during the COVID-19 pandemic compared to pre-pandemic. However, the magnitude of difference was relatively modest: the greatest relative decrease in prevalence was just 25% (for group sex). The overall prevalence of these behaviors remained surprisingly high – particularly for behaviors such as SOPV attendance and group sex – given the severity and longevity of pandemic lockdowns and physical distancing restrictions in Peru. Furthermore, for many of the sexual behaviors evaluated in this study (condomless anal sex, transactional sex, substance use in a sexual context), we found no significant difference in prevalence during the pandemic compared to pre-pandemic. These findings suggest that, while the COVID-19 pandemic led to some changes in how MSM in Lima met and formed sexual partnerships, its impact on other factors relevant to HIV vulnerability may have been negligible.

In addition, among MSM surveyed during the COVID-19 pandemic, we found that SOPV attendance was associated with several HIV-related sexual risk behaviors (e.g., more sexual partners, group sex, having a casual partner, and alcohol use in a sexual context). This mirrors the results of our previously published analysis of the association between SOPV attendance and sexual behaviors in this population in the pre-pandemic survey [24]. Similarly, in our analysis of factors associated with online platform use during the pandemic, MSM who reported meeting a recent partner online were more likely to report they engaged in all of the sexual behaviors evaluated in this study, consistent with findings from previous studies in this population and others demonstrating an association between online partner seeking and HIV-related risk behaviors [25, 33]. Despite the modest decrease in SOPV attendance and online platform use we observed among MSM in Lima during the pandemic, it is clear from our interaction term analysis that the strength of the associations with high-risk sexual behaviors was similar during and before the pandemic.

Approximately half of HIV-negative participants in the mid-pandemic survey reported they encountered challenges accessing HIV testing, consistent with previous studies of the impact of the COVID-19 pandemic on access to sexual health services among MSM in other settings [1–4]. While relatively few participants reported a history of current or prior PrEP use (consistent with the low availability of PrEP in Peru prior to mid-2023), nearly half of those who did reported disruptions to their PrEP use related to the pandemic. Among HIV-positive participants, a relatively lower proportion (33%) reported any pandemic-related disruption in treatment services, which may suggest prioritization of HIV treatment services over testing and prevention by healthcare systems during the pandemic. Interestingly, a greater number of participants rated their current physical and overall health as better or the same, rather than worse, compared to before the pandemic, yet the reverse was found when asking about mental health. Many previous studies examining the impact of the pandemic on mental health have observed a decrease in physical activity and an increase in stress and anxiety, likely due to social isolation [34–36]. These findings are important to highlight given the well-studied associations between poor mental health and risk factors for sexual HIV transmission, particularly among MSM [37–39]. Poorer mental health, coupled with the relatively high proportion of participants reporting barriers to accessing HIV services mid-pandemic – despite engaging in sexual risk behaviors at similar rates as participants pre-pandemic – raises important concerns about ensuring continuity of care for this key population, including a potentially greater need for mental health services among MSM. With almost 90% of participants in our study reporting that their ability to protect themselves from STIs was either better or the same during the pandemic, perhaps pandemic restrictions such as social distancing – which limited opportunities for sexual encounters – contributed to how participants perceived their overall and physical health. Despite most participants perceiving that they were less likely to engage in certain HIV-related sexual risk behaviors (e.g., CAS, substance use in a sexual context), we found no significant difference in the reported prevalence of these same behaviors when compared to the prevalence among our pre-pandemic sample of MSM in Lima, who were surveyed just prior to the onset of the COVID-19 pandemic.

There were several limitations to this study. The possibility of sampling bias was particularly relevant to our primary analytical objective, which involved comparing two separate convenience samples from the mid-pandemic and pre-pandemic time periods. This was necessary, given that the original study was not designed to re-sample the same group of participants longitudinally. Despite our best efforts to replicate the same online recruitment strategy and outreach methods for the mid-pandemic survey, a significantly higher proportion of participants in the mid-pandemic sample identified as bisexual and as HIV positive relative to the pre-pandemic sample. Stratifying by each of these variables did not reveal evidence of confounding that would alter our interpretation of the results (Supplementary Tables 1 and 2, Additional File 1). Furthermore, we used multivariable regression to control for differences between the two samples based on two key sociodemographic factors: age and educational attainment. The addition of HIV status and sexual orientation to these multivariable models yielded similar results. Nonetheless, we cannot rule out the possibility of residual confounding, which limits the strength of the conclusions that can be drawn about pandemic-related changes in behavior. Additionally, in both survey settings over half of participants were ≥ 30 years old, and close to half had a university degree. As such, it is unclear how generalizable our findings are to younger MSM in Peru, or those with lower educational attainment. Our anonymous online survey approach also meant that we could not employ certain data quality measures typical for online surveys; however, the fact that there was no compensation or incentive for survey participation serves to minimize the likelihood of duplicate or other spurious responses. It should also be noted that the sexual behaviors we assessed, including condomless anal sex, are relatively imprecise proxies for true HIV transmission or acquisition risk experienced by an individual, as these do not account for the potential use of powerful HIV prevention methods (e.g., PrEP, U = U, serosorting) by participants. Finally, Peru faced a surge in COVID-19 cases during the first half of 2021 [28], yet the majority of mid-pandemic participants responded in the second half of 2021 (Supplementary Fig. 1, Additional File 1). Because of changes in behavior over time during the course of the pandemic, survey response date could have influenced our prevalence estimates for the mid-pandemic survey. However, we performed a sensitivity analysis demonstrating the robustness of our findings across different time periods based on the response date of mid-pandemic survey participants over the 10 + month recruitment period (Supplementary Table 7, Additional File 1).

Our findings provide important new insights about the experiences of MSM in Peru during the COVID-19 pandemic and how the pandemic may have shaped HIV vulnerability. The relatively modest degree of change we observed in HIV-related sexual risk behaviors during the pandemic, including SOPV attendance and online platform use, underscore the need for sustained outreach to vulnerable populations such as MSM – even in the face of profound societal disruptions – in order to ensure access to HIV testing, treatment and prevention services. Future research and programmatic efforts are needed to better understand and address the barriers that MSM experience in accessing vital sexual health services, including support for re-engagement in HIV treatment and prevention services when interrupted by external forces such as a pandemic [40–42]. Given the increasing availability of PrEP in Peru since this study was conducted, additional research to understand factors influencing PrEP access and uptake will also be important in this setting to support ongoing implementation efforts. 

## Conclusion

Despite a decrease in some HIV-related sexual risk behaviors during the COVID-19 pandemic among MSM in Peru, the degree of change was relatively modest. Online platforms were still frequently used to facilitate sexual encounters, and SOPVs remained important spaces for partners to meet. Online platform use and SOPV attendance continued to be associated with a range of HIV-related sexual risk behaviors, mirroring findings from prior to the pandemic. Our findings illustrate the need for targeted public health interventions that address the unique challenges faced by MSM in accessing sexual health services, including HIV testing and treatment, including during a pandemic. Future efforts should focus on ensuring continuity of HIV prevention and sexual health services in the context of global health crises, as well as developing proactive strategies to mitigate the negative mental health impacts of pandemic-related disruptions and social isolation.

## Supplementary Information

Supplementary Material 1: Supplementary Figure 1. Histogram of survey responses by response date. Supplementary Table 1. Comparison of sexual behaviors and HIV testing/prevention knowledge during the mid-pandemic versus pre-pandemic period, stratified by HIV status (*N* = 769). Supplementary Table 2. Comparison of sexual behaviors and HIV testing/prevention knowledge during the mid-pandemic versus pre-pandemic period, stratified by sexual orientation (bisexual versus any other sexual orientation) (*N* = 769). Supplementary Table 3. Factors associated with SOPV attendance during the mid-pandemic period, stratified by HIV status (*N* = 387). Supplementary Table 4. Factors associated with SOPV attendance during the mid-pandemic period, stratified by sexual orientation (bisexual versus any other sexual orientation) (*N* = 387). Supplementary Table 5. Factors associated with online platform use during the mid-pandemic period, stratified by HIV status (*N* = 387). Supplementary Table 6. Factors associated with online platform use during the mid-pandemic period, stratified by sexual orientation (bisexual versus any other sexual orientation) (*N* = 387). Supplementary Table 7. Sexual behaviors and HIV testing/prevention knowledge during the mid-pandemic versus pre-pandemic period, by mid-pandemic survey period. Supplementary Table 8. Comparison of sexual behaviors and HIV testing/prevention knowledge during the mid-pandemic versus pre-pandemic period, further adjusting for sexual orientation, HIV status, and knowledge of U = U (*N* = 769).

## Data Availability

The datasets used and analyzed during the current study are available from the corresponding author upon reasonable request.

## Abbreviations

MSM: Men who have sex with men
SOPV: Sex-on-premises venue
GSN: Geosocial networking
CAS: Condomless anal sex
ART: Antiretroviral treatment
PrEP: Pre-exposure prophylaxis (for HIV)
PEP: Post-exposure prophylaxis (for HIV)
STI: Sexually transmitted infection
PR: Prevalence ratio
CI: Confidence interval
IQR: Interquartile range
U = U: Undetectable equals untransmissible
LSD: Lysergic acid diethylamide
