# Supplementary material for: Sexual behaviors and access to HIV services during the COVID-19 pandemic among cisgender men who have sex with men in Lima, Peru
Source: BMC Public Health. 2025 Aug 22;25:2888. doi: 10.1186/s12889-025-23886-8 (PMC12372404; doi:10.1186/s12889-025-23886-8)
Supplement: Supplementary file 1 — Supplementary Material 1: Supplementary Figure 1. Histogram of survey responses by response date. Supplementary Table 1. Comparison of sexual behaviors and HIV testing/prevention knowledge during the mid-pandemic versus pre-pandemic period, stratified by HIV status (N = 769). Supplementary Table 2. Comparison of sexual behaviors and HIV testing/prevention knowledge during the mid-pandemic versus pre-pandemic period, stratified by sexual orientation (bisexual versus any other sexual orientation) (N = 769). Supplementary Table 3. Factors associated with SOPV attendance during the mid-pandemic period, stratified by HIV status (N = 387). Supplementary Table 4. Factors associated with SOPV attendance during the mid-pandemic period, stratified by sexual orientation (bisexual versus any other sexual orientation) (N = 387). Supplementary Table 5. Factors associated with online platform use during the mid-pandemic period, stratified by HIV status (N = 387). Supplementary Table 6. Factors associated with online platform use during the mid-pandemic period, stratified by sexual orientation (bisexual versus any other sexual orientation) (N = 387). Supplementary Table 7. Sexual behaviors and HIV testing/prevention knowledge during the mid-pandemic versus pre-pandemic period, by mid-pandemic survey period. Supplementary Table 8. Comparison of sexual behaviors and HIV testing/prevention knowledge during the mid-pandemic versus pre-pandemic period, further adjusting for sexual orientation, HIV status, and knowledge of U = U (N = 769). [file 12889_2025_23886_MOESM1_ESM.docx]

**Supplementary Figure 1.** Histogram of survey responses by response date


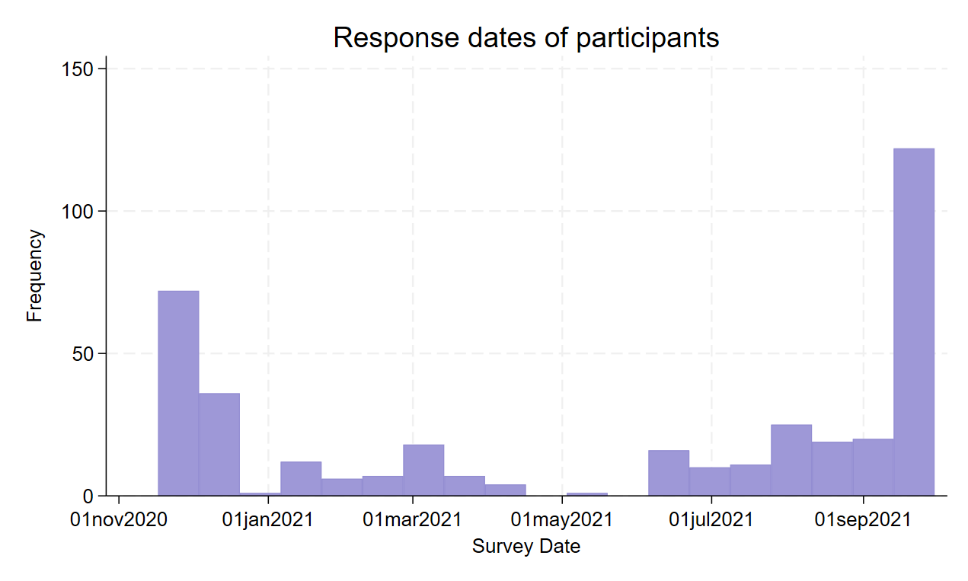


**Supplementary Table 1.** Comparison of sexual behaviors and HIV testing/prevention knowledge during the mid-pandemic versus pre-pandemic period, stratified by HIV status (N = 769)

|  | Crude PR (95% CI) | | | Multivariable PR (95% CI) | | |
| --- | --- | --- | --- | --- | --- | --- |
|  | Unstratified | Not bisexual | Bisexual | Unstratified | Not bisexual | Bisexual |
| Attended SOPV | **0.81 (0.72–0.91)** | 0.89 (0.78–1.02) | **0.68 (0.55–0.85)** | **0.82 (0.73–0.92)** | 0.90 (0.79–1.02) | **0.68 (0.55–0.85)** |
| Met partner online | **0.83 (0.76–0.91)** | 0.92 (0.83–1.02) | **0.67 (0.56–0.80)** | **0.84 (0.76–0.92)** | 0.94 (0.85–1.04) | **0.67 (0.56–0.80)** |
| ≥ 3 sexual partners | **0.85 (0.74–0.98)** | 0.92 (0.78–1.09) | **0.74 (0.57–0.97)** | **0.85 (0.73–0.98)** | 0.93 (0.78–1.10) | **0.71 (0.54–0.94)** |
| Casual partner | 0.97 (0.86–1.11) | 1.09 (0.94–1.26) | 0.79 (0.61–1.00) | 0.98 (0.87–1.12) | 1.10 (0.95–1.28) | 0.78 (0.61–1.00) |
| Group sex | **0.76 (0.61–0.94)** | 0.93 (0.71–1.22) | **0.50 (0.36–0.70)** | **0.75 (0.61–0.93)** | 0.92 (0.70–1.21) | **0.51 (0.37–0.71)** |
| Received payment for sex | 0.99 (0.70–1.39) | 0.91 (0.58–1.42) | 1.04 (0.60–1.82) | 1.02 (0.73–1.44) | 0.94 (0.60–1.45) | 1.09 (0.63–1.90) |
| Condomless anal sex | 0.96 (0.82–1.12) | 0.90 (0.75–1.10) | 1.10 (0.82–1.47) | 0.98 (0.83–1.14) | 0.94 (0.77–1.13) | 1.07 (0.80–1.43) |
| Any substance use in sexual context | 0.97 (0.82–1.14) | 1.02 (0.84–1.24) | 0.89 (0.66–1.19) | 0.98 (0.84–1.16) | 1.02 (0.84–1.24) | 0.91 (0.68–1.20) |
| Alcohol | 0.96 (0.79–1.16) | 1.05 (0.84–1.30) | 0.84 (0.58–1.22) | 0.97 (0.80–1.18) | 1.05 (0.84–1.31) | 0.86 (0.59–1.26) |
| Marijuana | 0.87 (0.62–1.20) | 0.75 (0.49–1.15) | 1.02 (0.59–1.75) | 0.87 (0.63–1.20) | 0.75 (0.50–1.14) | 1.05 (0.62–1.78) |
| Poppers | 0.94 (0.64–1.39) | 0.90 (0.51–1.59) | 0.81 (0.48–1.37) | 0.93 (0.63–1.37) | 0.87 (0.49–1.54) | 0.85 (0.51–1.43) |
| Ever heard of PrEP | 1.08 (1.00–1.17) | 1.06 (0.95–1.18) | 1.05 (0.94–1.17) | 1.05 (0.97–1.14) | 1.04 (0.93–1.15) | 1.05 (0.94–1.16) |
| Believes U=U | **1.23 (1.09–1.38)** | **1.28 (1.09–1.51)** | 1.02 (0.89–1.18) | **1.22 (1.08–1.37)** | **1.29 (1.10–1.52)** | 1.02 (0.89–1.18) |

Bold denotes statistical significance.

**Supplementary Table 2.** Comparison of sexual behaviors and HIV testing/prevention knowledge during the mid-pandemic versus pre-pandemic period, stratified by sexual orientation (bisexual versus any other sexual orientation) (N = 769)

|  | Crude PR (95% CI) | | | Multivariable PR (95% CI) | | |
| --- | --- | --- | --- | --- | --- | --- |
|  | Unstratified | HIV- | HIV+ | Unstratified | HIV- | HIV+ |
| Attended SOPV | **0.81 (0.72–0.91)** | **0.76 (0.67–0.87)** | 1.15 (0.90–1.47) | **0.82 (0.73–0.92)** | **0.78 (0.69–0.89)** | 1.11 (0.86–1.42) |
| Met partner online | **0.83 (0.76–0.91)** | **0.80 (0.73–0.89)** | 0.97 (0.76–1.24) | **0.84 (0.76–0.92)** | **0.81 (0.73–0.89)** | 1.01 (0.79–1.28) |
| ≥ 3 sexual partners | **0.85 (0.74–0.98)** | **0.82 (0.70–0.96)** | 1.09 (0.76–1.55) | **0.85 (0.73–0.98)** | **0.82 (0.70–0.96)** | 1.03 (0.73–1.47) |
| Casual partner | 0.97 (0.86–1.11) | 0.95 (0.83–1.09) | 1.06 (0.72–1.55) | 0.98 (0.87–1.12) | 0.96 (0.84–1.10) | 1.07 (0.73–1.56) |
| Group sex | **0.76 (0.61–0.94)** | **0.74 (0.59–0.92)** | 0.87 (0.47–1.59) | **0.75 (0.61–0.93)** | **0.73 (0.58–0.92)** | 0.79 (0.44–1.41) |
| Received payment for sex | 0.99 (0.70–1.39) | 1.00 (0.68–1.48) | 1.09 (0.53–2.24) | 1.02 (0.73–1.44) | 1.04 (0.71–1.53) | 1.06 (0.51–2.18) |
| Condomless anal sex | 0.96 (0.82–1.12) | 0.99 (0.84–1.18) | 0.78 (051–1.21) | 0.98 (0.83–1.14) | 1.03 (0.86–1.22) | 0.76 (0.49–1.16) |
| Any substance use in sexual context | 0.97 (0.82–1.14) | 0.98 (0.82–1.17) | 0.86 (0.54–1.37) | 0.98 (0.84–1.16) | 1.01 (0.84–1.20) | 0.86 (0.54–1.35) |
| Alcohol | 0.96 (0.79–1.16) | 0.96 (0.78–1.18) | 0.94 (0.57–1.55) | 0.97 (0.80–1.18) | 0.99 (0.81–1.22) | 0.92 (0.56–1.50) |
| Marijuana | 0.87 (0.62–1.20) | 0.93 (0.66–1.31) | 0.28 (0.06–1.20) | 0.87 (0.63–1.20) | 0.93 (0.66–1.30) | 0.30 (0.07–1.20) |
| Poppers | 0.94 (0.64–1.39) | 0.96 (0.64–1.44) | 0.51 (0.11–2.42) | 0.93 (0.63–1.37) | 0.95 (0.63–1.43) | 0.54 (0.11–2.52) |
| Ever tested for HIV | **1.09 (1.02–1.17)** | **1.09 (1.02–1.17)** |  | 1.07 (1.00–1.14) | 1.07 (1.00–1.14) |  |
| Tested for HIV last 6 months | **1.09 (1.02–1.17)** | 1.07 (1.00–1.15) | 1.12 (0.911.39) | 1.07 (1.00–1.14) | **1.05 (0.99–1.12)** | 1.08 (0.88–1.33) |
| Ever heard of PrEP | 0.89 (0.74–1.07) | 0.86 (0.71–1.05) | 1.03 (0.67–1.59) | 0.87 (0.73–1.04) | **0.85 (0.70–1.04)** | 0.97 (0.63–1.48) |
| Believes U=U | **1.23 (1.09–1.38)** | **1.18 (1.05–1.33)** | 1.27 (0.80–2.02) | **1.22 (1.08–1.37)** | **1.19 (1.05–1.33)** | 1.27 (0.80–2.03) |

Bold denotes statistical significance.

**Supplementary Table 3.** Factors associated with SOPV attendance during the mid-pandemic period, stratified by HIV status (N = 387)

|  | Crude PR (95% CI) | | | Multivariable PR (95% CI) | | |
| --- | --- | --- | --- | --- | --- | --- |
|  | Unstratified | HIV- | HIV+ | Unstratified | HIV- | HIV+ |
| ≥ 3 sexual partners | **2.00 (1.55–2.58)** | **1.95 (1.41–2.69)** | **1.97 (1.30–3.01)** | **2.02 (1.56–2.60)** | **1.92 (1.39–2.66)** | **2.07 (1.38–3.11)** |
| Casual partner | **1.94 (1.56–2.41)** | **1.83 (1.41–2.39)** | **1.97 (1.35–2.88)** | **1.92 (1.54–2.39)** | **1.80 (1.38–2.36)** | **2.01 (1.37–2.94)** |
| Group sex | **2.82 (1.87–4.25)** | **3.01 (1.71–5.32)** | **2.64 (1.45–4.82)** | **2.79 (1.85–4.22)** | **2.91 (1.63–5.18)** | **2.75 (1.52–4.98)** |
| Received payment for sex | **1.89 (1.11–3.23)** | 1.81 (0.84–3.90) | **2.21 (1.05–4.67)** | **1.74 (1.01–2.99)** | 1.66 (0.76–3.60) | 2.01 (0.95–4.28) |
|  |  |  |  |  |  |  |
| Condomless anal sex | 1.11 (0.88–1.40) | 1.35 (0.98–1.87) | 0.87 (0.61–1.25) | 1.11 (0.88–1.40) | 1.30 (0.94–1.81) | 0.91 (0.64–1.31) |
| Diagnosed w/STI | 1.20 (0.92–1.55) | 1.01 (0.70–1.45) | **1.59 (1.10–2.30)** | 1.26 (0.98–1.63) | 1.01 (0.71–1.45) | **1.71 (1.21–2.43)** |
| Any substance use in sexual context | **1.48 (1.16–1.90)** | 1.26 (0.93–1.71) | **1.89 (1.25–2.85)** | **1.50 (1.16–1.92)** | 1.26 (0.92–1.72) | **1.98 (1.32–2.98)** |
| Alcohol | **1.47 (1.10–1.95)** | 1.22 (0.87–1.70) | **1.91 (1.12–3.26)** | **1.44 (1.07–1.93)** | 1.18 (0.84–1.66) | **2.01 (1.16–3.46)** |
| Marijuana | 1.14 (0.70–1.85) | 0.86 (0.44–1.69) | 1.71 (0.85–3.44) | 1.28 (0.78–2.09) | 0.96 (0.48–1.93) | 1.91 (0.97–3.77) |
| Poppers | 1.19 (0.68–2.10) | 0.90 (0.38–2.17) | 1.76 (0.84–3.67) | 1.35 (0.77–2.39) | 1.01 (0.42–2.47) | 2.00 (0.98–4.07) |
| Ever heard of PrEP | 0.94 (0.85–1.04) | 0.96 (0.83–1.11) | 0.96 (0.85–1.09) | 0.99 (0.90–1.09) | 1.02 (0.88–1.18) | 0.98 (0.86–1.12) |
| Believes U=U | 0.96 (0.83–1.10) | 1.13 (0.90–1.41) | 0.86 (0.72–1.03) | 1.00 (0.87–1.15) | 1.15 (0.92–1.44) | 0.91 (0.76–1.08) |

Bold denotes statistical significance.

**Supplementary Table 4.** Factors associated with SOPV attendance during the mid-pandemic period, stratified by sexual orientation (bisexual versus any other sexual orientation) (N = 387)

|  | Crude PR (95% CI) | | | Multivariable PR (95% CI) | | |
| --- | --- | --- | --- | --- | --- | --- |
|  | Unstratified | Not bisexual | Bisexual | Unstratified | Not bisexual | Bisexual |
| ≥ 3 sexual partners | **2.00 (1.55–2.58)** | **2.00 (1.53–2.62)** | 1.85 (0.79–4.34) | **2.02 (1.56–2.60)** | **2.03 (1.55–2.65)** | 1.68 (0.68–4.13) |
| Casual partner | **1.94 (1.56–2.41)** | **2.05 (1.64–2.57)** | 1.27 (0.60–2.69) | **1.92 (1.54–2.39)** | **2.04 (1.62–2.56)** | 1.15 (0.53–2.49) |
| Group sex | **2.82 (1.87–4.25)** | **2.67 (1.77–4.04)** | – | **2.79 (1.85–4.22)** | **2.69 (1.77–4.08)** | – |
| Received payment for sex | **1.89 (1.11–3.23)** | 1.72 (0.97–3.03) | 3.18 (0.44–23.01) | **1.74 (1.01–2.99)** | 1.66 (0.93–2.96) | 2.42 (0.26–22.06) |
| Condomless anal sex | 1.11 (0.88–1.40) | 1.15 (0.90–1.46) | 0.92 (0.41–2.05) | 1.11 (0.88–1.40) | 1.14 (0.90–1.46) | 0.78 (0.33–1.83) |
| Diagnosed w/STI | 1.20 (0.92–1.55) | 1.15 (0.88–1.50) | 4.59 (0.66–32.11) | 1.26 (0.98–1.63) | 1.21 (0.93–1.58) | 3.32 (0.50–22.03) |
| Any substance use in sexual context | **1.48 (1.16–1.90)** | **1.50 (1.16–1.94)** | 1.65 (0.56–4.81) | **1.50 (1.16–1.92)** | **1.48 (1.14–1.92)** | 1.92 (0.78–4.72) |
| Alcohol | **1.47 (1.10–1.95)** | **1.47 (1.09–1.99)** | 1.53 (0.52–4.51) | **1.44 (1.07–1.93)** | **1.41 (1.04–1.92)** | 1.61 (0.65–3.98) |
| Marijuana | 1.14 (0.70–1.85) | 1.37 (0.84–2.26) | – | 1.28 (0.78–2.09) | 1.49 (0.90–2.47) | – |
| Poppers | 1.19 (0.68–2.10) | 1.35 (0.75–2.40) | 0.35 (0.02–5.37) | 1.35 (0.77–2.39) | 1.45 (0.81–2.60) | 0.95 (0.06–14.41) |
| Ever tested for HIV | 1.06 (0.96–1.16) | 0.99 (0.94–1.04) | 1.45 (0.97–2.19) | 1.08 (0.99–1.19) | 1.03 (0.94–1.12) | **1.96 (1.14–3.35)** |
| Tested for HIV last 6 months | 1.26 (0.95–1.71) | 1.22 (0.89–1.66) | 1.77 (0.65–4.84) | 1.30 (0.96–1.74) | 1.22 (0.89–1.67) | 1.91 (0.74–4.95) |
| Ever heard of PrEP | 0.94 (0.85–1.04) | 0.98 (0.89–1.08) | 0.84 (0.51–1.39) | 0.99 (0.90–1.09) | 1.01 (0.92–1.12) | 0.96 (0.58–1.59) |
| Currently on PrEP | 2.10 (0.80–5.56) | 2.66 (0.91–7.74) | 0.39 (0.03–5.85) | 2.30 (0.85–6.24) | 2.82 (0.94–8.45) | 0.23 (0.05–1.00) |
| Believes U=U | 0.96 (0.83–1.10) | 0.96 (0.83–1.10) | 2.00 (0.70–5.70) | 1.00 (0.87–1.15) | 0.98 (0.85–1.13) | 2.19 (0.74–6.46) |

Bold denotes statistical significance.

**Supplementary Table 5.** Factors associated with online platform use during the mid-pandemic period, stratified by HIV status (N = 387)

|  | Crude PR (95% CI) | | | Multivariable PR (95% CI) | | |
| --- | --- | --- | --- | --- | --- | --- |
|  | Unstratified | HIV- | HIV+ | Unstratified | HIV- | HIV+ |
| ≥ 3 sexual partners | **3.69 (2.51–5.43)** | **2.59 (1.66–4.05)** | **6.33 (3.07–13.04)** | **3.73 (2.54–5.48)** | **2.61 (1.67–4.07)** | **6.13 (2.98–12.61)** |
| Casual partner | **2.85 (2.11–3.86)** | **2.36 (1.63–3.41)** | **3.54 (2.11–5.92)** | **2.84 (2.10–3.83)** | **2.32 (1.61–3.37)** | **3.54 (2.12–5.91)** |
| Group sex | **3.08 (1.89–5.03)** | **3.45 (1.66–7.17)** | **2.84 (1.45–5.57)** | **3.09 (1.90–5.02)** | **3.50 (1.71–7.14)** | **2.87 (1.45–5.67)** |
| Received payment for sex | **2.24 (1.20–4.19)** | **1.61 (0.69–3.78)** | **3.58 (1.42–9.01)** | **2.27 (1.22–4.22)** | **1.67 (0.74–3.78)** | **3.70 (1.46–9.39)** |
|  |  |  |  |  |  |  |
| Condomless anal sex | **1.34 (1.03–1.74)** | 1.16 (0.82–1.64) | **1.67 (1.13–2.46)** | **1.34 (1.03–1.74)** | 1.13 (0.80–1.60) | **1.63 (1.11–2.39)** |
| Diagnosed w/STI | **1.52 (1.12–2.05)** | 1.51 (0.96–2.39) | **1.70 (1.14–2.54)** | **1.57 (1.18–2.10)** | **1.59 (1.03–2.46)** | **1.63 (1.10–2.40)** |
| Any substance use in sexual context | **1.77 (1.32–2.37)** | **1.94 (1.27–2.97)** | **1.58 (1.04–2.42)** | **1.76 (1.31–2.36)** | **1.97 (1.29–3.00)** | **1.62 (1.08–2.44)** |
| Alcohol | **1.64 (1.18–2.28)** | **1.82 (1.17–2.84)** | 1.25 (0.74–2.12) | **1.63 (1.18–2.27)** | **1.84 (1.18–2.88)** | 1.28 (0.76–2.13) |
| Marijuana | **2.92 (1.48–5.78)** | **3.62 (1.13–11.57)** | **2.98 (1.27–6.99)** | **2.96 (1.48–5.91)** | **3.87 (1.18–12.69)** | **3.03 (1.33–6.91)** |
| Poppers | **2.47 (1.18–5.16)** | 3.42 (0.81–14.45) | **2.70 (1.14–6.39)** | **2.43 (1.14–5.16)** | 3.37 (0.74–15.33) | **2.80 (1.23–6.36)** |
| Ever heard of PrEP | 1.01 (0.91–1.13) | 1.15 (0.95–1.38) | 0.95 (0.84–1.07) | 1.02 (0.92–1.13) | 1.16 (0.98–1.39) | 0.95 (0.84–1.06) |
| Believes U=U | 1.11 (0.95–1.30) | **1.37 (1.04–1.81)** | 1.01 (0.85–1.21) | 1.11 (0.95–1.29) | **1.37 (1.04–1.81)** | 1.01 (0.85–1.19) |

Bold denotes statistical significance.

**Supplementary Table 6.** Factors associated with online platform use during the mid-pandemic period, stratified by sexual orientation (bisexual versus any other sexual orientation) (N = 387)

|  | Crude PR (95% CI) | | | Multivariable PR (95% CI) | | |
| --- | --- | --- | --- | --- | --- | --- |
|  | Unstratified | Not bisexual | Bisexual | Unstratified | Not bisexual | Bisexual |
| ≥ 3 sexual partners | **3.69 (2.51–5.43)** | **2.93 (2.20–3.91)** | 1.50 (0.94–2.39) | **3.73 (2.54–5.48)** | **2.94 (2.21–3.93)** | **1.68 (1.08–2.60)** |
| Casual partner | **2.85 (2.11–3.86)** | **2.29 (1.82–2.89)** | 1.70 (1.00–2.87) | **2.84 (2.10–3.83)** | **2.29 (1.82–2.88)** | 1.71 (1.00–2.90) |
| Group sex | **3.08 (1.89–5.03)** | **3.13 (2.12–4.62)** | 1.30 (0.65–2.59) | **3.09 (1.90–5.02)** | **3.12 (2.11–4.61)** | 1.51 (0.78–2.95) |
| Received payment for sex | **2.24 (1.20–4.19)** | **2.54 (1.42–4.56)** | 2.07 (0.76–5.64) | **2.27 (1.22–4.22)** | **2.58 (1.45–4.60)** | 2.24 (0.85–5.90) |
|  |  |  |  |  |  |  |
| Condomless anal sex | **2.85 (2.11–3.86)** | **1.42 (1.14–1.77)** | 1.28 (0.79–2.07) | **1.34 (1.03–1.74)** | **1.40 (1.12–1.74)** | 1.39 (0.87–2.21) |
| Diagnosed w/STI | **1.52 (1.12–2.05)** | **1.64 (1.19–2.27)** | 0.79 (0.32–1.95) | **1.57 (1.18–2.10)** | **1.69 (1.24–2.31)** | 0.96 (0.40–2.32) |
| Any substance use in sexual context | **1.77 (1.32–2.37)** | **1.69 (1.33–2.14)** | 1.53 (0.86–2.73) | **1.76 (1.31–2.36)** | **1.66 (1.31–2.11)** | 1.62 (0.92–2.85) |
| Alcohol | **1.64 (1.18–2.28)** | **1.48 (1.14–1.92)** | 1.52 (0.82–2.83) | **1.63 (1.18–2.27)** | **1.46 (1.12–1.89)** | 1.64 (0.89–3.01) |
| Marijuana | **2.92 (1.48–5.78)** | **3.97 (2.12–7.45)** | – | **2.96 (1.48–5.91)** | **3.89 (2.07–7.30)** | – |
| Poppers | **2.47 (1.18–5.16)** | **3.71 (1.82–7.55)** | 2.90 (0.37–22.90) | **2.43 (1.14–5.16)** | **3.62 (1.76–7.44)** | 2.69 (0.31–23.23) |
| Ever tested for HIV | **1.16 (1.02–1.31)** | **1.16 (1.03–1.32)** | 1.11 (0.75–1.65) | **1.16 (1.03–1.31)** | **1.17 (1.03–1.32)** | 1.17 (0.82–1.68) |
| Tested for HIV last 6 months | 1.30 (0.93–1.81) | 1.30 (1.00–1.69) | 1.05 (0.70–1.55) | 1.30 (0.93–1.81) | 1.29 (0.99–1.68) | 1.12 (0.75–1.65) |
| Ever heard of PrEP | 1.01 (0.91–1.13) | 1.07 (0.96–1.19) | **0.64 (0.41–0.99)** | 1.02 (0.92–1.13) | 1.08 (0.97–1.20) | **0.62 (0.40–0.95)** |
| Currently on PrEP | 1.55 (0.62–3.83) | 1.38 (0.55–3.47) | – | 1.55 (0.64–3.75) | 1.35 (0.55–3.32) | – |
| Believes U=U | 1.11 (0.95–1.30) | 1.11 (0.95–1.29) | 1.31 (0.59–2.93) | 1.11 (0.95–1.29) | 1.11 (0.95–1.29) | 1.34 (0.59–3.03) |

Bold denotes statistical significance.

**Supplementary Table 7.**  **S**exual behaviors and HIV testing/prevention knowledge during the mid-pandemic versus pre-pandemic period, by mid-pandemic survey period

|  | **PR (95% CI)** | | | |
| --- | --- | --- | --- | --- |
|  | **Mid-Pandemic**  **Overall, n=382** | **Mid-Pandemic Period 1 (11/17/20 to 12/31/20), n=109** | **Mid-Pandemic Period 2 (1/1/21 to 7/31/21), n=110** | **Mid-Pandemic Period 3 (8/1/21 to 10/1/21), n=168** |
| Attended SOPV | **0.81 (0.72–0.91)** | **0.73 (0.60–0.90)** | **0.77 (0.63–0.93)** | 0.89 (0.77–1.02) |
| Met partner online | **0.83 (0.76–0.91)** | **0.73 (0.61–0.87)** | **0.85 (0.74–0.98)** | **0.88 (0.78–0.99)** |
| ≥ 3 sexual partners | **0.85 (0.74–0.98)** | **0.71 (0.55–0.92)** | 0.83 (0.66–1.04) | 0.96 (0.81–1.14) |
| Casual partner | 0.97 (0.86–1.11) | **0.75 (0.59–0.95)** | 1.04 (0.87–1.25) | 1.07 (0.92–1.25) |
| Group sex | **0.76 (0.61–0.94)** | **0.69 (0.48–0.98)** | 0.88 (0.65–1.19) | **0.72 (0.54–0.97)** |
| Received payment for sex | 0.99 (0.70–1.39) | 0.50 (0.25–1.02) | 1.18 (0.73–1.90) | 1.18 (0.78–1.77) |
| Condomless anal sex | 0.96 (0.82–1.12) | 0.97 (0.76–1.23) | 1.12 (0.90–1.38) | 0.85 (0.68–1.06) |
| Any substance use in sexual context | 0.97 (0.82–1.14) | 0.9 (0.69–1.16) | 1.07 (0.86–1.35) | 0.95 (0.77–1.17) |
| Alcohol | 0.96 (0.79–1.16) | 0.83 (0.6–1.13) | 1.04 (0.79–1.37) | 0.99 (0.78–1.26) |
| Marijuana | 0.87 (0.62–1.20) | 1.19 (0.77–1.83) | 0.85 (0.52–1.42) | 0.66 (0.41–1.07) |
| Poppers | 0.94 (0.64–1.39) | 1.14 (0.66–1.97) | 1.13 (0.66–1.95) | 0.69 (0.39–1.22) |
| Ever tested for HIV | **1.13 (1.05–1.22)** | **1.10 (1.04–1.18)** | **1.09 (1.02–1.17)** | **1.11 (1.06–1.18)** |
| Tested for HIV last 6 months | 0.86 (0.73–1.01) | **0.63 (0.43–0.91)** | 0.80 (0.61–1.06) | 0.99 (0.83–1.20) |
| Believes U=U | 1.10 (1.00–1.20) | **1.14 (1.02–1.28)** | 1.09 (0.95–1.24) | 1.07 (0.95–1.20) |

Bold denotes statistical significance

**Supplementary Table 8.** Comparison of sexual behaviors and HIV testing/prevention knowledge during the mid-pandemic versus pre-pandemic period, further adjusting for sexual orientation, HIV status, and knowledge of U=U (N = 769)

|  | Multivariable PR (95% CI) | | |
| --- | --- | --- | --- |
|  | Original (Age & Education) | With Sexual Orientation & HIV Status | With Sexual Orientation, HIV Status, & U=U Knowledge |
| Attended SOPV | **0.82 (0.73–0.92)** | **0.83 (0.74–0.93)** | **0.83 (0.74–0.93)** |
| Met partner online | **0.84 (0.76–0.92)** | **0.84 (0.77–0.92)** | **0.84 (0.76–0.92)** |
| ≥ 3 sexual partners | **0.85 (0.73–0.98)** | **0.86 (0.74–0.99)** | **0.85 (0.73–0.98)** |
| Casual partner | 0.98 (0.87–1.12) | 0.99 (0.87–1.12) | 0.98 (0.86–1.12) |
| Group sex | **0.75 (0.61–0.93)** | **0.73 (0.59–0.91)** | **0.72 (0.58–0.90)** |
| Received payment for sex | 1.02 (0.73–1.44) | 1.01 (0.72–1.41) | 1.013 (0.72–1.43) |
| Condomless anal sex | 0.98 (0.83–1.14) | 0.98 (0.83–1.15) | 0.97 (0.83–1.14) |
| Any substance use in sexual context | 0.98 (0.84–1.16) | 0.98 (0.83–1.16) | 0.98 (0.83–1.15) |
| Alcohol | 0.97 (0.80–1.18) | 0.99 (0.82–1.20) | 0.99 (0.81–1.20) |
| Marijuana | 0.87 (0.63–1.20) | 0.84 (.61–1.16) | 0.83 (0.60–1.15) |
| Poppers | 0.93 (0.63–1.37) | 0.85 (0.58–1.25) | 0.81 (0.55–1.19) |
| Ever tested for HIV^a^ | 1.07 (1.00, 1.14) | 1.06 (0.99, 1.13) | 1.04 (0.98, 1.11) |
| Tested for HIV last 6 months^a^ | 0.91 (0.76, 1.09) | 0.91 (0.76, 1.09) | 0.88 (0.74, 1.06) |
| Ever heard of PrEP | 1.05 (0.97–1.14) | 1.04 (0.93–1.15) | 1.05 (0.94–1.16) |
| Believes U=U | **1.22 (1.08–1.37)** | **1.16 (1.03–1.30)** | – |

Bold denotes statistical significance; a. Among N = 270 pre-pandemic and N = 226 mid-pandemic HIV-negative participants and model excludes sexual orientation covariable
